# New Scaffolds of Proteasome Inhibitors: Boosting Anticancer Potential by Exploiting the Synergy of In Silico and In Vitro Methodologies

**DOI:** 10.3390/ph16081096

**Published:** 2023-08-02

**Authors:** Romina A. Guedes, Jorge H. Grilo, Andreia N. Carvalho, Pedro M. P. Fernandes, Ana S. Ressurreição, Vanessa Brito, Adriana O. Santos, Samuel Silvestre, Eleonora Gallerani, Maria João Gama, Riccardo Gavioli, Jorge A. R. Salvador, Rita C. Guedes

**Affiliations:** 1 Research Institute for Medicines (iMed.ULisboa), Faculty of Pharmacy, University of Lisbon, 1649-003 Lisboa, Portugal or rominaguedes@ff.uc.pt (R.A.G.); jorge.grilo@campus.ul.pt (J.H.G.); amcarvalho@ff.ulisboa.pt (A.N.C.); pfernandes@cnc.uc.pt (P.M.P.F.); aressurreicao@ff.ulisboa.pt (A.S.R.); mjgama@ff.ulisboa.pt (M.J.G.); 2 Center for Innovative Biomedicine and Biotechnology (CIBB), Center for Neuroscience and Cell Biology (CNC), University of Coimbra, 3004-504 Coimbra, Portugal; 3 Laboratory of Pharmaceutical Chemistry, Faculty of Pharmacy, University of Coimbra, 3000-548 Coimbra, Portugal; 4 Health Sciences Research Centre (CICS-UBI), University of Beira Interior, 6200-506 Covilhã, Portugal; vanessa.sofia.brito@ubi.pt (V.B.); aos@ubi.pt (A.O.S.); sms@ubi.pt (S.S.); 5 Department of Chemical, Pharmaceutical and Agricultural Sciences, University of Ferrara, 44121 Ferrara, Italy; eleonora.gallerani@unife.it

**Keywords:** ubiquitin–proteasome system, proteasome, proteasome inhibitors, cancer, leukemia, lymphoma, multiple myeloma, molecular docking, virtual screening

## Abstract

Cancer is a complex multifactorial disease whose pathophysiology involves multiple metabolic pathways, including the ubiquitin–proteasome system, for which several proteasome inhibitors have already been approved for clinical use. However, the resistance to existing therapies and the occurrence of severe adverse effects is still a concern. The purpose of this study was the discovery of novel scaffolds of proteasome inhibitors with anticancer activity, aiming to overcome the limitations of the existing proteasome inhibitors. Thus, a structure-based virtual screening protocol was developed using the structure of the human 20S proteasome, and 246 compounds from virtual databases were selected for in vitro evaluation, namely proteasome inhibition assays and cell viability assays. Compound **4** (JHG58) was shortlisted as the best hit compound based on its potential in terms of proteasome inhibitory activity and its ability to induce cell death (both with IC_50_ values in the low micromolar range). Molecular docking studies revealed that compound **4** interacts with key residues, namely with the catalytic Thr1, Ala20, Thr21, Lys33, and Asp125 at the chymotrypsin-like catalytic active site. The hit compound is a good candidate for additional optimization through a hit-to-lead campaign.

## 1. Introduction

Cancer is a multifactorial disease and is the second leading cause of mortality and morbidity after heart diseases and, according to the World Health Organization (WHO), it is predicted to affect 22 million people by 2030 [1].

Eukaryotic cells have two major pathways for intracellular protein degradation: the lysosomal pathway and the ubiquitin–proteasome system (UPS). The proteasome is the most relevant proteolytic system and exerts a key role in the ATP-dependent controlled protein degradation, namely in carcinogenesis [2,3,4,5,6,7,8,9,10]. Substrates of the proteasome include signaling molecules, tumor suppressors (e.g., p53) and promoters, cell cycle regulators (e.g., cyclins), transcription factors [e.g., c-Jun, nuclear factor kappa B (NF-κB), and E2F1], and anti-apoptotic proteins (e.g., Bcl-2), among others [5,11]. When a disruption in the degradation of these protein substrates occurs, the consequences are very deleterious, especially in cancer cells, which depend more on the proteasome to remove misfolded or damaged proteins as a result of their genetic instability and the rapid proliferation rate associated with an accelerated and uncontrolled mitosis [12,13,14,15,16,17].

**Figure 1:**
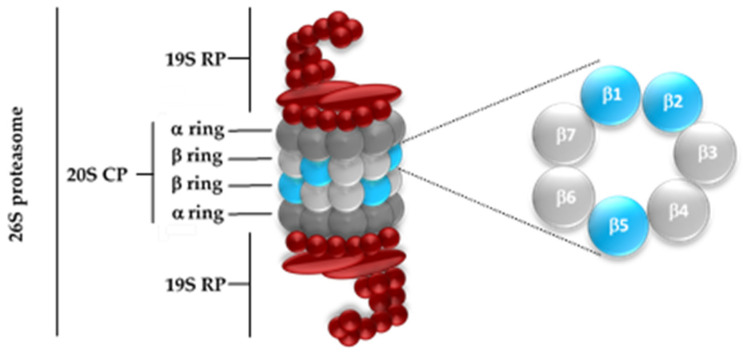
Structure of the eukaryotic 26S proteasome. The 26S proteasome is composed of one 20S CP and two 19S RPs. The catalytic center is located in the β rings, which have three catalytic subunits with different cleavage patterns: β1 (C-L), β2 (T-L), and β5 (CT-L).

The 26S proteasome (Figure 1) has a molecular weight of about 2500 kilodaltons (kDa) and includes a 20S core particle (CP) and two 19S regulatory particles (RPs) [7,18,19,20,21]. The 20S CP (usually known as “20S proteasome” or just as “proteasome”) represents the catalytic portion of the proteasomal system [22].

The inner β rings are responsible for the proteolytic activity. However, in eukaryotic proteasomes, only three out of the seven different β subunits have catalytic activity: β1, β2, and β5 (Figure 1). Each of the three proteolytic subunits has differences with respect to both binding as well as activity performed: the β1 subunit has caspase-like (C-L) activity or “post acidic” (PA) and cleaves peptide bonds after acidic amino acids; the β2 subunit exerts a trypsin-like (T-L) activity, cleaving peptide bonds after basic amino acids; the β5 subunit presents chymotrypsin-like (CT-L) activity and acts after neutral and hydrophobic amino acids [19,23,24,25,26,27]. This is possible because the catalytic β subunits have a threonine 1 (Thr1) N-terminal, in which the γ hydroxyl group (Thr1Oγ) acts as a nucleophile in the hydrolysis of the peptide bond [5,20,23,28,29,30,31,32,33].

The results of site-directed mutagenesis in yeast revealed that the CT-L sites are the most important in protein degradation and various synthetic proteasome inhibitors are usually optimized according to their capacity to block the β5 active sites [16]. However, several of the most potent proteasome inhibitors described in the literature also inhibit the C-L and/or the T-L active sites (bortezomib has an IC_50_ of 7 nM for the CT-L active site and an IC_50_ of 74 nM for the C-L activity [34], ixazomib has an IC_50_ of 3.4 nM for the chymotryptic activity and an IC_50_ of 31 nM for the C-L active site [35], and marizomib inhibits the CT-L active site with an IC_50_ of 3.5 nM and and T-L activity IC_50_ of 28 nM [36]). Thus, it may be worth exploring molecules that can interact with more than one catalytic site. 

**Figure 2:**
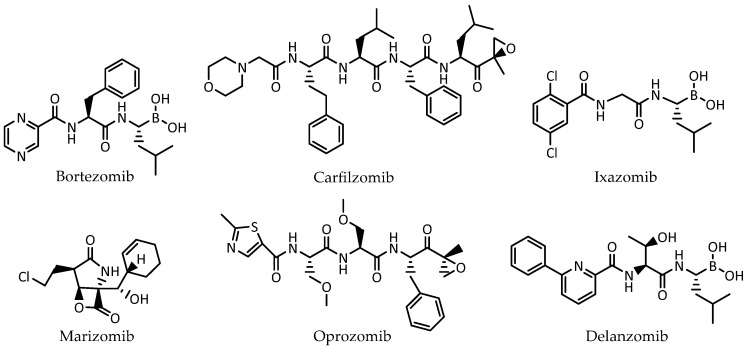
The six proteasome inhibitors on the market or clinically used: bortezomib, carfilzomib, ixazomib, marizomib, oprozomib, and delanzomib.

Six proteasome inhibitors are on the market or have been in clinical use: bortezomib, carfilzomib, ixazomib, marizomib, delanzomib, and oprozomib (Figure 2). Bortezomib has marketing authorization for the treatment of multiple myeloma and mantle cell lymphoma, while carfilzomib and ixazomib are only approved for the treatment of multiple myeloma [37,38,39,40].

Despite its slowly reversible mode of action, the formation of a metastable but long-lasting covalent inhibitor-proteasome adduct causes an unfavorable pharmacodynamic profile since a large fraction of the intravenously administrated dose is lost due to the inhibition of proteasomes in non-tumor tissues, (e.g., red blood cells, liver, and the vascular endothelium). While irreversible proteasome blockage is desirable and effective when targeting parasites, it should not be a primary choice in anticancer therapy or immune diseases therapy due to off-target effects (e.g., peripheral neuropathy) and dose-limiting cytotoxicity against healthy cells. Furthermore, cellular defense mechanisms induce mutations and the overexpression of the proteasome following treatment with irreversible or slowly reversible inhibitors, therefore leading to resistance [33]. On the other hand, reversible and hence time-limited proteasome inhibition without employing a reactive electrophilic warhead is suggested to increase target selectivity, thus avoiding the intrinsic drawbacks associated with either irreversible or slowly reversible adduct formation [33].

The aim of this work was the discovery of novel proteasome inhibitor scaffolds with anticancer activity that can advance to a hit-to-lead campaign. For this, through the combination of computer-aided drug design (CADD) methodologies and in vitro assays, new scaffolds of proteasome inhibitors were identified from virtual libraries. A hit compound, herein designated compound **4**, was selected based on its proteasome inhibitory activity and effect on cell viability. Furthermore, molecular docking studies showed that compound **4** interacts with relevant residues, namely with the catalytic Thr1, Ala20, Thr21, Lys33, and Asp125 at the CT-L active site, making it a promising candidate.

## 2. Results and Discussion

In order to select potential proteasome inhibitors and evaluate their anticancer activity, the structure-based virtual screening (SBVS) of several virtual databases was performed, followed by in vitro evaluation (proteasome inhibition assays and cell viability assays).

### 2.1. Structure-Based Virtual Screening

#### 2.1.1. Databases 

A multistep SBVS campaign was undertaken in parallel for the three proteasomal active sites to identify new proteasome inhibitor scaffolds. Noncovalent docking using GOLD software (ChemPLP [41] scoring function) was performed on a total of 268,551 compounds assembled from diverse databases: National Cancer Institute (NCI) Open Database [42] (265,242 compounds), DrugBank 4.0 [43] (Approved subset, 1822 compounds), and an our in-house database (1487 compounds).

#### 2.1.2. Compound Selection 

The SBVS results were filtered according to several parameters: the docking score value, the molecular descriptor signature of the proteasome inhibitors of the CT-L active site, visual inspection, and the presence of possible promiscuous compounds. 

The first filter considered for compound selection was the docking score. While validating the docking protocol, among the most active compounds (IC_50_ between 3.4 and 47 nM) [34,35,44,45,46,47,48,49,50], bortezomib had the lowest score values at the three catalytic sites (CT-L = 73.55, C-L = 77.85, T-L = 72.94—Table S1) and these were used to set the cut-off limits for its respective active site (CT-L = 73, C-L = 77, T-L = 72). An exception was made for the in-house database cut-off value (60) in order to fully explore the chemical space.

Then, molecular descriptors were applied: molecular weight (MW, 350–650), violations of Lipinski’s Rule of 5 (Ro5, 0–2), hydrogen bond donors (HBD, 2–5), and logP ≥ 0. These ranges were established by considering the “average values” of the existing compounds available on the market/for clinical use. For example, an MW of 350–650 has been set because ixazomib, bortezomib, carfilzomib, marizomib, and oprozomib have, respectively, MW values of 361.03, 384.24, 719.92, 313.78, and 532.62 g.mol^−1^; the MW of carfilzomib has not been defined as an upper limit because potential hit compounds can be structurally modified in the optimization step, which may lead to an increase in the MW value. Selected compounds were allowed up to two violations of Lipinski’s Ro5 as about 6% of orally administrated drugs are known to be in the “beyond rule of 5 (bRo5)” chemical space. Currently, 21% of the new molecules approved by the FDA are bRo5 compounds, which also include antineoplastic drugs [51].

Sometimes, the inadequate sampling of the conformational space for the ligand may lead, for example, to unrealistic poses and intraligand steric clashes, which might lead to high score values. Therefore, a visual inspection of the docking poses was performed with MOE software to select the most suitable compounds to proceed for biological assays [52,53].

Finally, we evaluated the possible existence of compounds that can interfere in biological assays, namely because of non-specific inhibition (“aggregators”) or generating false assay readouts (“Pan-assay interference compounds—PAINS”). However, this filter was considered more as an “alert” and not a direct exclusion factor since some compounds in clinical use have structural alerts in their scaffold (e.g., carfilzomib and oprozomib epoxide groups).

In the selection process of the NCI database, among the 265,242 compounds, around 25,000 compounds had a score above 73; after the application of the other filters (and also considering the NCI compounds that were effectively available), 40 compounds were selected for in vitro evaluation. For the Approved dataset of DrugBank, among the one thousand eight hundred and twenty two compounds, one hundred and forty seven compounds had the desired score, seventy nine passed the molecular descriptors filter, and twenty nine of these compounds showed an interesting interaction profile; however, only six were acquired for biochemical evaluation. For the in-house database (1487 molecules), 361 compounds had a score above 73 for the CT-L active site and 286 compounds passed the molecular descriptors filter, from which 200 showed to interact with relevant residues and so were selected for in vitro assays. 

**Figure 3:**
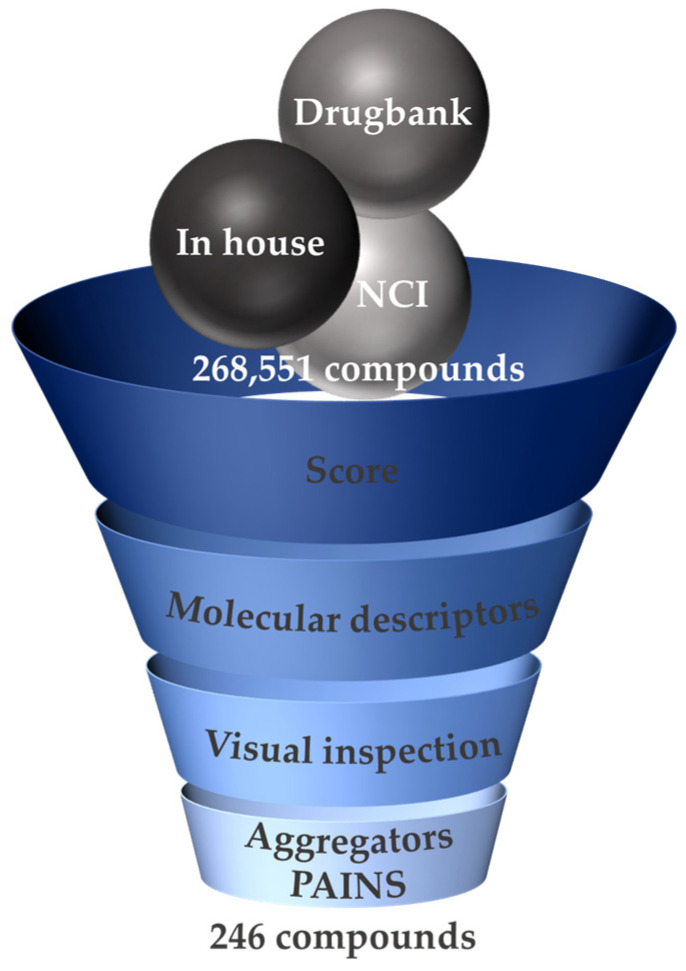
The general SBVS workflow. The SBVS of several databases was performed through noncovalent docking (GOLD 5.2, ChemPLP scoring function). Out of 268,551 compounds, 246 were selected for in vitro evaluation.

In summary, a total of 246 compounds (two hundred from the in-house database, six from DrugBank, and forty from the NCI) were selected for in vitro assays (Figure 3).

**Figure 4:**
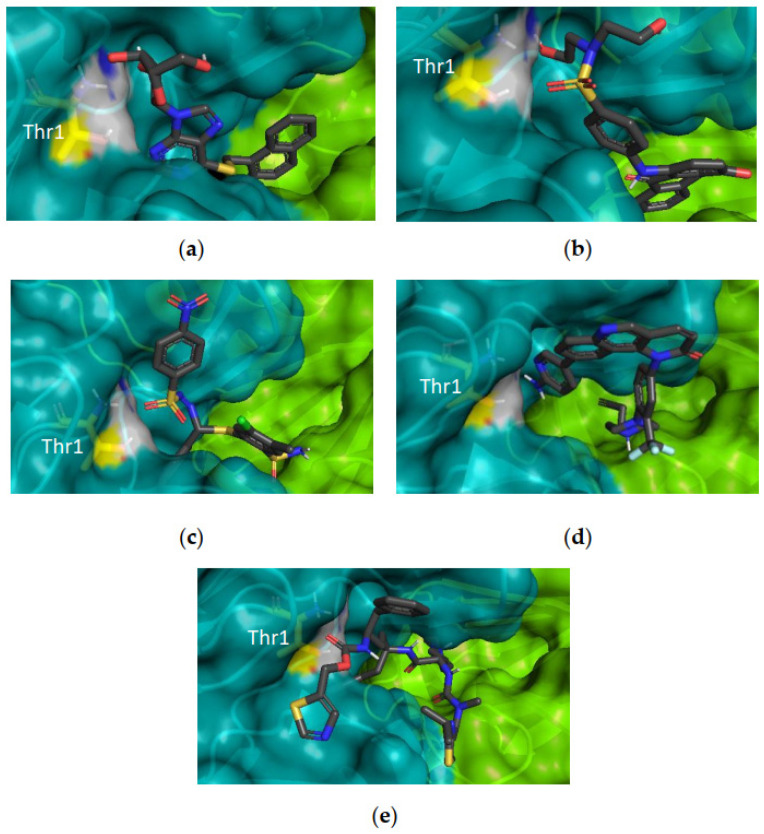
Some examples of compounds identified using SBVS docked to the CT-L active site. (**a**) NSC55467 (score = 76.87); (**b**) NSC115290 (score = 79.26); (**c**) NSC728499 (score = 76.66); (**d**) JHG58 (score = 69.24); (**e**) Cobicistat (score = 112.53). Blue surface: β5; green surface: β6; Thr1: yellow surface.

A sample of compounds selected for in vitro assays and their respective score values for the CT-L active site can be found in Figure 4 (scores for the three active sites can be found in Table S2). It is possible to observe that these compounds are well positioned in the binding pocket, being close to the catalytic Thr1. Moreover, it is interesting to observe that cobicistat (a compound from the Approved dataset of DrugBank) is the one with the highest score value.

### 2.2. In Vitro Studies

#### 2.2.1. Proteasome Inhibition Assays in Isolated Human 20S Proteasomes

To assess the inhibitory potential of the compounds selected through the SBVS campaign, two hundred and forty six compounds were tested in isolated human 20S proteasomes, with an initial screening being performed for the three active sites. In this assay, the peptide substrates are bound to aminoluciferin (Suc-LLVY-AML for CT-L activity, Z-nLPnLD-AML for C-L, and Z-LRR-AML for T-L), leading the cleavage of these substrates by the proteasome to the release of free aminoluciferin, which is the substrate of the buffer-contained luciferase [54,55].

**Figure 5:**
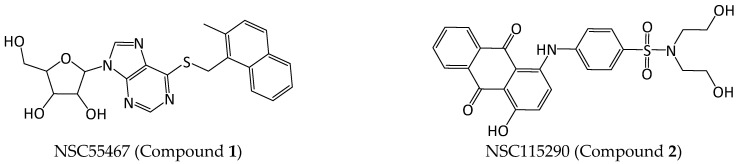

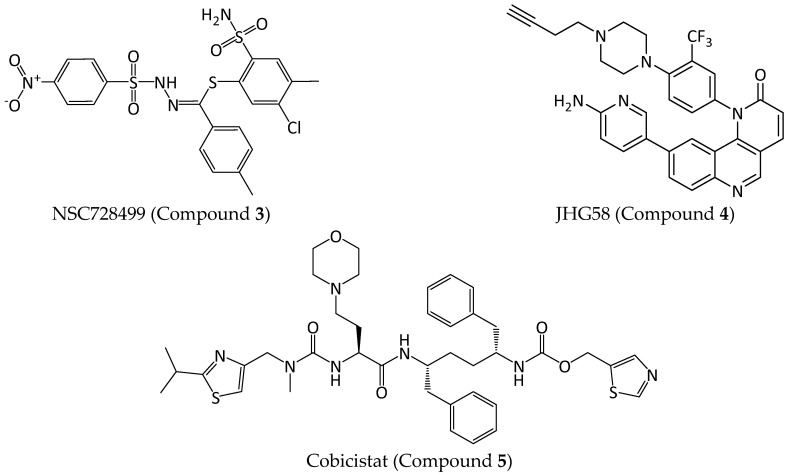
Compounds selected after inhibition assays in isolated human 20S proteasomes.

Table 1 (percentages, standard deviation (SD) values, and *p* values can be found in Table S3) lists the five compounds that showed an inhibition of at least approximately 40%: compounds **1**–**3** (NSC55467, NSC115290, and NSC728499, respectively) were selected from the NCI database, compound **4** (JHG58) is an in-house compound, and compound **5** (cobicistat) is from DrugBank (Figure 5). Compounds **2**–**4** showed a stronger inhibitory potential of the three activities; for this reason, additional concentrations were tested and IC_50_ values were determined. In isolated human 20S proteasomes, compound **2** showed the highest inhibitory potential, with IC_50_ values in the low micromolar range, especially in CT-L (5.60 µM) and C-L (2.42 µM) activities, while also exerting inhibition of the T-L activity (25.84 µM).

#### 2.2.2. Proteasome Inhibition Assays in Cell Lysates

After the assessment of the inhibitory activity of the selected compounds in isolated 20S proteasomes, it was important to evaluate their inhibitory potential in proteasomes from different cancer cell lines. Compounds **1**–**5** were tested in semi-purified proteasomes from cell lysates of human hematological and solid cancer cell lines (Jurkat, K562, HT-29, and HCT116) (Table 2; percentages, SD, and *p* values can be found in Supplementary Material—Tables S4–S7). The results revealed some differences when compared with assays in isolated human 20S proteasomes.

In fact, for most of the compounds, the IC_50_ values obtained in isolated proteasomes tend to be lower. For example, in compound **2**, the difference is significant: 5.60 µM in isolated 20S proteasomes versus 73.15 µM for the CT-L active site in Jurkat cells. A possible explanation for this difference might be the potential interaction of the compounds with other potential targets that might be present in the semi-purified proteasomes (cell lysates).

While compounds **1**, **3**, and **4** showed a slightly higher inhibitory potential of the C-L activity than of the CT-L activity in isolated proteasomes (e.g., compound **4** showed IC_50_ values of 48.36 µM and 51.13 µM in the C-L and CT-L active sites, respectively), in cancer cell lines, most of the compounds showed a stronger inhibition of the CT-L active site, followed by the C-L and T-L active sites (bortezomib, the positive control, shows the same inhibition profile, which is aligned with Demo et al. [34]); actually, most of the compounds did not show significant inhibition of the tryptic activity. 

Moreover, some compounds showed a higher inhibition of the chymotryptic activity in the lysates of solid cancer cell lines than in the ones of hematological cancers, e.g., compounds 1 (CT-L: 54% in Jurkat, 63% in K562, 62% in HT-29, 90% in HCT116) and 5 (CT-L: 64% in Jurkat, 81% in HT-29, 67% in HCT116). However, despite these results, it is important to consider that, in vivo, this pattern might change because, usually, proteasome inhibitors have a limited success in clinical trials for solid tumors largely due to poor tissue penetration as well as a rapid clearance from the bloodstream [56,57].

Despite compounds **2** and **3** showing a stronger inhibitory potential in isolated 20S proteasomes (especially compound **2**, with IC_50_ values in the low micromolar range for the CT-L and C-L activities—5.60 and 2.42 µM, respectively), compound **4** was the strongest inhibitor in semi-purified proteasomes, e.g., in Jurkat, cells the IC_50_ values were of 24.25 µM and 27.05 µM for the CT-L and C-L activities, respectively; furthermore, compound **4** IC_50_ values of the CT-L (51.13 µM) and C-L (41.18 µM) activities of isolated proteasomes were higher than those in semi-purified proteasomes, although no inhibition of the T-L activity was observed in cancer cells.

#### 2.2.3. Cell Viability Assays 

To study the effects of the compounds on cell viability, the 3-(4,5-dimethylthiazol-2-yl)-2,5-diphenyltetrazoliumbromide (MTT) assay was performed in the human cancer cell lines treated with the selected compounds at 100 µM (Table 3; percentages, SD, and *p* values can be found in Supplementary Material—Tables S8 and S9). Whenever possible, additional concentrations were tested and IC_50_ values were determined.

Compound **4** showed the highest cytotoxic effect in the tested cell lines, especially in Jurkat (IC_50_ = 1.37 µM) and HCT116 (IC_50_ = 1.02 µM). Regarding the compounds from the NCI database, compound **1** had a stronger cytotoxic effect in all cell lines. Compound **5** also affected cell viability, especially in Jurkat cells (IC_50_ = 43.83 µM).

#### 2.2.4. Hit Compound Selection and Docking Studies

The results obtained using SBVS workflow and confirmed by biological assays led us to select compound **4** as our best candidate (hit compound). This compound inhibited isolated human 20S proteasomes (IC_50_ values: CT-L = 51.13 µM, C-L = 41.18 µM, T-L = 48.36 µM) and, in semi-purified proteasomes from cancer cells, we observed an improvement of the IC_50_ values, especially in Jurkat cells (CT-L = 24.25 µM, C-L = 27.05 µM). Furthermore, the inhibition of the C-L active site in HCT116, with an IC_50_ of 28.91 µM, should be highlighted. Additionally, compound **4** also had the highest cytotoxic effect in the tested cell lines, affecting cell viability particularly in Jurkat cells (IC_50_ = 1.37 µM) and in the HCT116-cell line (IC_50_ = 1.02 µM).

**Figure 6:**
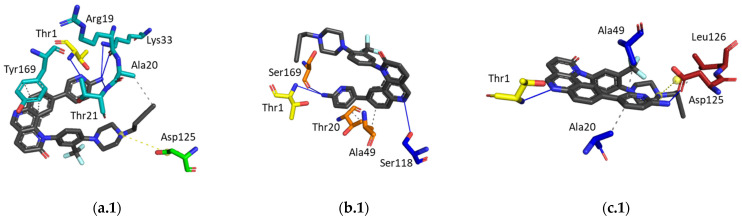

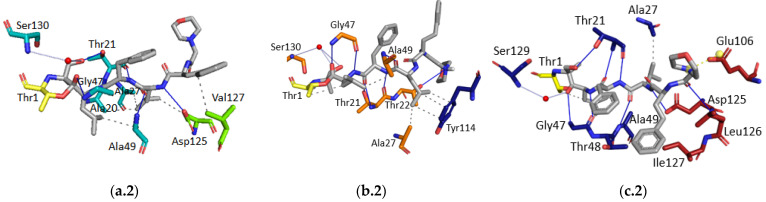
Ligand interactions of compound **4** (dark grey) and ligand interactions of the crystallographic ligand (carfilzomib, light grey) at the three active sites. For compound **4**, noncovalent ligand interactions are represented, while the crystallographic ligand interacts with the active site through covalent (with the catalytic Thr1) and noncovalent bonds. (**a.1**,**a.2**), CT-L active site; (**b.1**,**b.2**), C-L active site; (**c.1**,**c.2**), T-L active site. Light blue: β5; green: β6; orange: β1; dark blue: β2; red: β3; yellow: Thr1; blue line: hydrogen bond; white line: water bridge; dashed grey line: hydrophobic interaction; dashed yellow line and yellow sphere: salt bridge and charged center; red sphere: water molecule (present in the crystal structure, PDB ID 4R67).

Docking studies (Figure 6) allow us to understand the mode of binding of compound **4**. However, to analyze the relevance of the existent interactions, it is important to consider the available data described in the literature.

Huber et al. [58] described the most important amino acids for each proteasome active site. In eukaryotes, the most relevant active site amino acids are Thr1, Asp17, Lys33, Ser129, Asp166, and Ser169. Thr1, Asp17, and Lys33 are considered the most important residues in the proteolytic mechanism, while Ser129, Asp166, and Ser169 contribute not only for catalysis, but also for active site structural integrity [8,58,59].

Moreover, in the publication where the crystal structure used for our calculations is described, Harshbarger et al. [60] remark that the substrate selectivity for each catalytic site is determined by the interaction of the substrate’s P1 side chain with the active site’s S1 specificity pocket. In particular, residue 45 at the base of the S1 pocket is important in the determination of the three distinct cleavage preferences. In the CT-L active site, the β5 subunit amino acids of the S1 pocket responsible for the chymotryptic activity are Ala20, Met45, Ala49, and Cys52; Met45 enables the accommodation of hydrophobic residues. Regarding the C-L active site, the S1 pocket includes the residues Thr20, Thr31, Thr35, Arg45, Ala49, Gln53, and β2Asp120, with the positively charged Arg45 at the base of the S1 pocket, which allows for the accommodation of acidic residues and performance of the C-L activity. Finally, the T-L activity of subunit β2 enables cleaving after basic residues due to the acidic character created by Cys31, His35, and Asp53 residues from β2, and to the Cys129 from β3. The T-L active site has an S1 pocket that includes a Gly45 residue, creating an S1 pocket larger than the existing one in the other two active sites [60].

Through the observation of the X-ray structure used for the docking calculations (Figure 6), beyond the covalent bond with the catalytic Thr1, carfilzomib interacts with several residues. At the CT-L active site, carfilzomib interacts with Ala20, Thr21, Ala27, Gly47, Ala49, Asp125, Val 127, and Ser130; at the C-L active site, the crystal ligand interacts with Thr21, Thr22, Ala27, Gly47, Ala49, Tyr114, and Ser130; finally, at the T-L active site, this ligand interacts with the residues Thr21, Ala27, Gly47, Thr48, Ala49, Glu106, Asp125, Leu126, Ile127, and Ser129.

Considering the docking studies of compound **4**, we can observe interactions with relevant residues of the CT-L active site, namely the catalytic Thr1, Lys33, important amino acids from the S1 specificity pocket (Ala20 and Thr21), and also with the Asp125, which means it has four interactions that were also observed in the X-ray. At the C-L active site, the hit compound owes its inhibitory potential mainly due to the interaction with the catalytic Thr1, with the S1 pocket residues Thr20 and Ala49, and also with Ser169, having two interactions in common with the crystal carfilzomib. Finally, the docking results reveal that the T-L activity is mostly inhibited through the interaction of compound **4** with Thr1, Ala20, Ala49, and Asp125, meaning four common interactions with the crystal ligand were observed (Thr1, Ala49, Asp125, and Leu126).

## 3. Materials and Methods 

### 3.1. In Silico Studies 

#### 3.1.1. Protein Structure Preparation

The receptor structure was retrieved from the Protein Data Bank (PDB) through the PDB code 4R67 (“Human constitutive 20S proteasome in complex with carfilzomib”, 2.89 Å). To prepare the enzyme structure of the three active sites, all atoms other than the receptor β5 and β6 subunits (chains L and M, respectively—CT-L active site), β1 and β2 subunits (chains H and I, respectively—C-L active site), and β2 and β3 subunits (chains I and J, respectively—T-L active site) were deleted from the crystal structure using MOE software package (versions 2014.09 to 2019.0102). AMBER99 forcefield was used to assign atom types and charges to each atom in the protein. Hydrogen atoms were added and the protonation states were assigned using the Protonate-3D tool (pH = 7.4 and T = 310.15 K) within MOE software package (versions 2014.09 to 2019.0102). The protonation of the residues at the binding site was established according to the X-ray diffraction study performed by Harshbarger et al. [60].

#### 3.1.2. Databases Preparation

The structure of the compounds was retrieved from PubChem, from the respective databases or drawn in MOE (versions 2014.09 to 2019.0102). All the compounds included in this study were previously protonated with MOE’s Protonate-3D tool (pH = 7.4, 310.15 K) and partial charges were assigned using MMFF94x forcefield as implemented in the MOE software package (versions 2014.09 to 2019.0102). Databases were washed to remove salts and metals. The geometry and energy of all compounds gathered from the databases was minimized after protonation.

#### 3.1.3. Molecular Docking for Structure-based Virtual Screening and for the Compounds Evaluated in Biological Assays

Noncovalent docking calculations were performed with ChemPLP scoring function using the GOLD 5.2.0 suite of programs (Hermes version 1.6) [61,62,63,64]. The binding site was defined to be centered on the Thr1Oγ with a 15 Å radius. For SBVS, the number of GA runs was set to 100, a search efficiency of 30% was set, and the three top-ranked solutions for each ligand were selected. For compounds evaluated in biological assays, additional docking calculations were performed using 1000 GA runs, at 100% search efficiency, and the ten top-ranked solutions for each ligand were selected. The “early termination” and “save lone pairs” options were disabled. For the remaining settings, the default parameters were used. The final scores were ranked and the results were analyzed in MOE software package (versions 2014.09 to 2019.0102).

#### 3.1.4. Ligand Interaction Patterns

##### Noncovalent Interaction Patterns for Individual Ligands

Protein and ligand structures were prepared according to the previously mentioned methodology (MOE software) and saved in PDB format. The X-ray structures of each active site were downloaded from the PDB and the protein–ligand complexes were evaluated in MOE software (versions 2014.09 to 2019.0102). Noncovalent ligand interactions were analyzed in the PLIP v1.1.11 web server.

#### 3.1.5. Assessment of the Presence of Potential Aggregators and PAINS

The structures of the selected compounds were merged into a single sdf file with MOE software package and the file was uploaded to online tools. The presence of possible aggregators was assessed with the “Aggregator Advisor” [65] (Schoichet Laboratory, available: http://advisor.bkslab.org/, accessed from May 2018 to March 2019). FAF-Drugs4 (Free ADME-Tox Filtering Tool, available: http://fafdrugs4.mti.univ-paris-diderot.fr, accessed from May 2018 to March 2019) [66] was used to filter possible PAINS in the final subset of in silico selected compounds.

### 3.2. Preparation of 9-(6-Aminopyridin-3-yl)-1-(4-(4-(but-3-yn-1-yl)piperazin-1-yl)-3-(trifluoromethyl)phenyl)benzo[h][1,6]naphthyridin-2(1H)-one (JHG58, Compound **4**)

*Tert-butyl* 4-(4-(9-(6-aminopyridin-3-yl)-2-oxobenzo[*h*][1,6]naphthyridin-1(2*H*)-yl)-2-(trifluoromethyl)phenyl)piperazine-1-carboxylate [58] (23 mg, 0.045 mmol) was dissolved in a mixture of TFA (100 Eq) and dry DCM (1:1, *v*/*v*) and was left to stir, at room temperature, during 2 h. The reaction mixture was concentrated under reduced pressure and residual TFA was removed using successive dissolutions, followed by evaporation, with methanol (3×), toluene, and ether. The corresponding TFA salt was obtained as a white powder and was used in the next step without further purification. K_2_CO_3_ (40 mg, 0.287 mmol, 6.5 Eq) was added to a solution of the TFA salt in dry DMF (0.15 M) followed by propargyl bromide (0.053 mmol, 1.2 Eq). The reaction mixture was then stirred at 55 °C, under N_2_ atmosphere, over 12 h before being diluted with EtOAc. The organic phase was subsequently washed with water (3 × 10 mL) and brine and dried with anhydrous Na_2_SO_4_. The solvent was evaporated under reduced pressure and the crude product was purified using preparative TLC (DCM/MeOH, 97:3) to afford JHG58 as a pale yellow powder (12 mg, 48% over 2 steps).

TLC (DCM/MeOH = 97:3): Rf = 0.35 [UV]. ^1^H NMR (300 MHz, CDCl_3_) δ 8.96 (s, 1H), 8.15 (d, *J* = 8.7 Hz, 1H), 7.99 (d, *J* = 9.4 Hz, 1H), 7.92 (d, *J* = 2.5 Hz, 1H), 7.79 (dd, *J* = 8.7, 1.9 Hz, 1H), 7.65 (br. s, 1H), 7.52 (br. s, 2H), 7.14 (dd, *J* = 8.5, 2.5 Hz, 1H), 6.98 (d, *J* = 1.9 Hz, 1H), 6.94 (d, *J* = 9.4 Hz, 1H), 6.44 (d, *J* = 8.5 Hz, 1H), 4.54 (s, 2H), 3.15–3.03 (m, 2H), 3.01–2.91 (m, 2H), 2.79–2.65 (m, 6H), 2.46 (td, *J* = 7.5, 2.6 Hz, 2H), 2.02 (t, *J* = 2.6 Hz, 1H). ^13^C NMR (75 MHz, CDCl_3_) δ 164.3, 156.7, 149.7, 149.4, 146.2, 140.2 (q, ^3^*J*CF = 4.0 Hz), 138.2, 136.3, 135.6, 135.2, 134.3, 132.5, 129.9, 128.3, 127.3, 123.8, 123.5 (q, ^1^*J*CF = 268 Hz), 122.7, 122.3, 120.7, 120.1 (q, ^2^*J*CF = 34 Hz), 118.8, 114.3, 107.9, 84.2, 68.6, 53.9, 52.3, 50.1, 49.7, 48.3, 13.3. HRMS (ESI) *m/z* calcd for [C_32_H_28_F_3_N_6_O]+: 569.2271 [M + H]+; found 569.2297 (Figures S1 and S2).

### 3.3. In Vitro Assays

#### 3.3.1. Proteasome Inhibition Assays in Isolated 20S Proteasomes

To evaluate human purified erythrocyte-derived 20S proteasome inhibitory activity of the chosen compounds, the Proteasome-Glo™ 3-substrate system (Promega Corporation, Madison, WI, USA) was employed. The Proteasome-Glo™ reagents of the three proteolytic activities were prepared according to the manufacturer’s instructions, aliquoted, and stored at either −20 °C or 4 °C, avoiding freezing–thaw cycles. The screening for the inhibition of the three proteolytic activities of proteasome was carried out at the final concentrations of 10 µM and 100 µM for each of the tested compounds in 100 µL of reaction. The purified 20S proteasome (Enzo Life Sciences, Inc., Farmingdale, NY, USA) was diluted in HEPES buffer 10 mM, pH 7.4, to a concentration experimentally adjusted to the signal/noise ratio obtained with each particular lot of Proteasome-Glo™ reagent, in order to have a good compromise between sensitivity and linear response. Protein concentration was determined using the Pierce™ BCA protocol (Thermo Fisher Scientific, Rockford, IL, USA). All assays were performed in white-walled multiwell plates (Thermo Scientific™ Sterilin™ White Microtiter™ plates, Thermo Fisher Scientific, Paisley, UK) in duplicates. The proteasome solution (25 μL) was added to each well first, then the diluted solutions of the assayed compounds (25 μL), and lastly the Proteasome-Glo™ Reagent (50 μL). Each plate included the following controls: two wells of blank (no proteasome and no test compounds); at least four wells of the positive control (two with DMSO at 1%, two without DMSO; DMSO: 99.9% purity; Fisher Scientific, Loughbourough, UK); and at least one known inhibitory concentration of the reference compound, bortezomib. Reagents were added to the plate well on ice, and the plate was then transferred to the plate luminometer (SpectraMax^®^, Molecular Devices, LLC, San Jose, CA, USA) set at 37 °C, gently mixed for 30 s, and the measurements were immediately started in kinetic mode, with data collected every 5 min for at least 60 min.

All compounds were dissolved in DMSO at a concentration of 10 mM and stored at −20 °C or 4 °C. Solutions of the test compounds were prepared at their adequate dilutions in 10 mM HEPES buffer pH 7.6 before each experiment. The maximum DMSO concentration in each well was ≤1% (*v*/*v*), which guarantees no significant interference on cell proliferation.

To calculate the proteasome activity over time, the value of mean relative luminescence units (RLU) of blank wells at each respective time point was subtracted, and then the ratio between mean RLU of the sample and mean RLU of the positive control wells was calculated for each time point and expressed as percentage. The average proteasome activity at 60 min was considered in an overall comparison of the inhibitors, after being converted to percentage of proteasome inhibition and, whenever possible, expressed as IC_50_ by plotting the inhibition data of each compound at different concentrations against the log of the concentration of the inhibitor, using the GraphPad Prism software version 7. At least eight different concentrations of the test compounds were used. A minimum of three independent assays were performed for each sample and the results were displayed as mean ± SD.

#### 3.3.2. Cell Culture

Human leukemia Jurkat E6.1 T-cell line and K562-cell line were originally obtained from ATCC Collections (Manassas, VA, USA); human HT-29 and HCT116 colorectal carcinoma-cell lines were obtained from ECACC (Porton Down, Porton, UK). Cells were passaged for less than 6 months after and routinely tested for mycoplasma contamination using Mycoalert detection kit (Lonza, Basel, Switzerland). Jurkat, K562, and HT-29 cells were maintained in Roswell Park Memorial Institute (RPMI) 1640 with GlutaMAX™, supplemented with 1% antibiotic mixture (100 µg/mL streptomycin and 100 U/mL penicillin) and 10% heat-inactivated foetal bovine serum (FBS) (all from Gibco, Thermo Fisher Scientific, Paisley, UK). HCT116 cells were maintained in Dulbecco’s Modified Eagle Medium (DMEM) with GlutaMAX™, supplemented with 1% antibiotic mixture (100 µg/mL streptomycin and 100 U/mL penicillin) and 10% heat-inactivated FBS (all from Gibco, Thermo Fisher Scientific, Paisley, UK). Incubation was performed at 37 °C in a 5% CO_2_ atmosphere. Cells were routinely passaged every 2 or 3 days at 70–90% confluence; TrypLE Express (Gibco, Thermo Fisher Scientific, Paisley, UK) was used as dissociation agent for adherent cells. Trypan blue (Gibco, Thermo Fisher Scientific, Paisley, UK) was used to assess cell viability using the dye exclusion test.

#### 3.3.3. Proteasome Inhibition Assay in Cell Lysates

Cells were obtained from several cultured cell lines: Jurkat, K562, HT-29, and HCT116. Pellets with 20 × 10^6^ cells were prepared and washed with cold sterile phosphate buffered saline (PBS), 1×, pH 7.4 (Gibco, Thermo Fisher Scientific, Paisley, UK). The preparation of the lysates from several cell lines was performed based on the methodology described by Gavioli et al. [67]. Cells were resuspended in buffer containing 50 mM TRIS-HCl pH 7.4 (Nzytech, Lisboa, Portugal), 5 mM MgCl_2_ (≥98% purity, Merck, Hamburg, Germany), 1 mM DTT (Merck, Hamburg, Germany), 2 mM ATP (≥98% purity, Enzo Life Sciences Inc., Farmingdale, NY, USA), and 250 mM saccharose (Merck, Hamburg, Germany). Glass beads (Merck, Hamburg, Germany) equivalent to the volume of the cell suspension were added, and the mixture was vortexed for 2 min at 4 °C. Beads and cell debris were removed using 5 min centrifugation at 1000× *g* and 4 °C, followed by 20 min of centrifugation at 10,000× g at 4 °C. Cell lysates containing proteasomes were resuspended in “activity buffer” [50 mM TRIS-HCl (pH 7.4), 5 mM MgCl_2_, 1 mM DTT, 2 mM ATP (≥98% purity, Enzo Life Sciences Inc., Farmingdale, NY, USA), and 500 µM EDTA (≥98% purity, Merck, Hamburg, Germany)]. Protein concentration was determined using the Pierce™ BCA protocol (Thermo Fisher Scientific, Rockford, IL, USA). Stock solutions (10 mM) of each compound were made in DMSO (99.9% purity; Fisher Scientific, Loughbourough, UK) and diluted in activity buffer to give the concentrations to be tested. Bortezomib (98% purity, eNovation Chemicals, Bridgewater, NJ, USA) was used as positive control. DMSO was added in an equal volume as used for the untreated compounds as a way to assess/control its influence in the assay. Untreated cells were used as control. In each well of a 96-well plate, 25 µL of lysate were seeded in duplicate with 25 µL of the compound to test; in the control, 25 µL of activity buffer were added to the 25 µL of lysate. A blank (50 µL of activity buffer) was added to the wells in quadruplicate. Cell lysates, pre-treated or not with different concentrations of the test compounds, were incubated for 30 min at 37 °C. Suc-Leu-Leu-Val-Tyr-AMC (≥97% purity, Enzo Life Sciences Inc., Farmingdale, NY, USA), Z-Leu-Leu-Glu-AMC (≥95% purity, Enzo Life Sciences Inc., Farmingdale, NY, USA), and Boc-Leu-Arg-Arg-AMC (≥97% purity, Enzo Life Sciences Inc., Farmingdale, NY, USA) were used as substrates to measure CT-L, C-L, and T-L proteasome activities, respectively. Stock solutions (10 mM) of each substrate were prepared in DMSO, diluted in activity buffer, and 50 µL were added to all wells (final concentration of each substrate = 100 µM), followed by an incubation at 37 °C for 60 min. Samples fluorescence was measured at an excitation wavelength of 360 nm and emission of 465 nm; the fluorescence measures were performed using the GloMax-Multi + Detection System (Promega Corp., Madison, WI, USA). Activity was evaluated in fluorescence units, after being converted to percentage of proteasome inhibition and, whenever possible, expressed as IC_50_ by plotting the inhibition data of each compound at different concentrations against the log of the concentration of the inhibitor, using the GraphPad Prism software version 7. At least eight different concentrations of the test compounds were used. A minimum of three independent assays were performed for each sample and the results were displayed as mean ± SD.

#### 3.3.4. Assessment of Cell Viability using the MTT Assay

To assess cell viability, the MTT assay was performed. Jurkat/K562/HT-29/HCT116 cell lines were seeded in triplicate (10,000 cells/well) in 96-well plates with 50 µL of complete medium and incubated at 37 °C, 5% CO_2_ for 24 h to allow the cells to adhere to the plate (in the case of adherent cells). Stock solutions (10 mM) of each compound were prepared in DMSO (99.9% purity; Fisher Scientific, Loughbourough, UK) and diluted in complete medium to give the different dilutions to be tested. Bortezomib (98% purity, eNovation Chemicals) was used as positive control and DMSO was added in an equal volume of the compounds to assess its influence in the assay. Untreated cells seeded in triplicate were used as control. The blank was performed in triplicate with 100 µL of culture medium. The cells were incubated with the compounds, in triplicate, in 100 µL of total volume, at 37 °C, 5% CO_2_ for 72 h. A solution of MTT (98% purity; Sigma-Aldrich, St Louis, MO, USA) was prepared: MTT was dissolved in sterile PBS 1× pH 7.4 (Gibco, Thermo Fisher Scientific, Paisley, UK) to 5 mg/mL; the solution was filter-sterilized through a 0.2 µM filter into a sterile, light-protected container; the MTT solution must be stored protected from light at 4 °C for frequent use, or at −20 °C for long term storage. In suspension cells (Jurkat), 25 µL of MTT were directly added to each well and, after 3 h 30 m of incubation, 100 µL of DMSO were added to solubilize the formazan crystals; in adherent cells (HT-29 and HCT116), the culture medium was removed, 25 µL of MTT were added to each well and, after 3 h 30 m of incubation, upon MTT removal, 100 µL of DMSO were added to solubilize the formazan crystals. After 15 min, the samples absorbance was measured at 570 nm and the percentage of cell viability was determined. The measures were performed using the GloMax-Multi + Detection System (Promega Corp., Madison, WI, USA). Whenever possible, the IC_50_ was calculated by plotting the percentage of cell viability at different concentrations against the log of the concentration of the inhibitor, using the GraphPad Prism software version 7. At least eight different concentrations of the test compounds were used. A minimum of three independent assays were performed for each sample and the results were displayed as mean ± SD.

## 4. Conclusions

Cancer affects millions of people all over the world and is considered the second leading cause of mortality and morbidity after heart diseases.

The proteasome is considered an important target for cancer treatment and represents an intensive focus in this field of research. Currently, three proteasome inhibitors are on the market, but the resistance phenomena and the adverse effects reported are a serious concern. For this reason, the identification of new and selective proteasome inhibitors remains an important strategy in cancer treatment in order to provide safer and effective treatments.

Our study implemented and validated a molecular docking protocol to perform an SBVS workflow of commercial, approved, and in-house databases for a total of 268,551 compounds. Docking studies illustrated the interactions of the compounds with important residues in each active site, namely Thr1, Ala20, Ala22, Lys33, and Ala49, allowing for the explanation of their putative mechanisms of action; 246 compounds were then selected to proceed to in vitro assays.

The compounds were assessed through proteasome inhibition assays (in isolated human 20S proteasomes and in semi-purified proteasomes from human cancer cell lines) and cell viability assays (MTT), as these studies mainly focused on compounds **1**–**5**. Compound **4** was selected as a hit compound because of its inhibitory potential in isolated 20S proteasomes (IC_50_: CT-L = 51.13 µM, C-L = 41.18 µM, T-L = 48.36 µM) and semi-purified proteasomes of cancer cell lines (IC_50_: Jurkat—CT-L = 24.25 µM, C-L = 27.05 µM; HT-29—CT-L = 31.30 µM, C-L = 35.23 µM; HCT116—CT-L = 35.72 µM, C-L = 28.91 µM). Moreover, compound 4 also showed cytotoxic activity in the tested cell lines (Jurkat—IC_50_ = 1.37 µM, HCT116—IC_50_ = 1.02 µM).

The biological assays confirmed the identification of new proteasome inhibitor scaffolds. Considering that the compounds currently on the market or that are/have been in clinical use (bortezomib, carfilzomib, ixazomib, marizomib, delanzomib, and oprozomib), with the exception of marizomib which is a β-lactone, the other inhibitors belong to different classes (boronates and epoxyketones) but they have in common the existence of a peptide scaffold.

In future work, compound **4** should proceed to a hit-to-lead campaign in order to improve its inhibitory and cytotoxic potential, including the optimization of time/dose exposure in both tumoral and normal cells, prior to in vivo evaluation in animal models, which will also allow for the study of better routes of administration and delivery methods to ensure a targeted administration, as far as possible.

These results demonstrate the complexity of the proteasome as a target for anticancer therapy through an important multidisciplinary approach to optimize and speed up this process.

## Figures and Tables

**Table 1:** Inhibitory potential of the selected compounds in the isolated human 20S proteasome. The results are expressed as a percentage of proteasome inhibition of the CT-L/C-L/T-L activity, respectively, at 100 µM. For the strongest inhibitors, IC_50_ values were also determined. Bortezomib (10 µM) was used as a positive control. Data presented are mean triplicate value ± SD of at least three independent experiments. Statistical analysis was carried out using one-way ANOVA with Dunnett’s multiple comparisons test. Percentages, SD, and *p* values can be found in Table S3.

	Inhibition of the Isolated Human 20S Proteasome		
Compound ID	CT-L	C-L	T-L
**1**	38%	55%	52%
**2**	98% 5.60 ± 2.56 µM	98% 2.42 ± 4.74 µM	100% 25.84 ± 4.11 µM
**3**	91% 49.26 ± 4.55 µM	88% 51.14 ± 2.94 µM	95% 27.52 ± 3.78 µM
**4**	70% 51.13 ± 2.66 µM	78% 41.18 ± 1.76 µM	74% 48.36 ± 2.63 µM
**5**	36%	34%	5%
Bortezomib	100% 12.90 ± 1.53 nM	93% 168.80 ± 1.46 nM	34% 1.89 ± 1.74 µM

**Table 2:** Inhibitory potential of the selected compounds in lysates of human cancer cells. The results are expressed as percentage of proteasome inhibition of the CT-L/C-L/T-L activity at 100 µM. For the strongest inhibitors, IC_50_ values were determined. Bortezomib (10 µM) was used as positive control. Data presented are mean triplicate value ± SD of at least three independent experiments. Statistical analysis was carried out using one-way ANOVA with Dunnett’s multiple comparisons test. Percentages, SD, and *p* values can be found in Supplementary Material—Tables S4–S7). NI = No inhibition; ND = Not determined.

		Inhibition of the 20S Proteasome in Cell Lysates of Human Cancer Cells			
		Cell Line			
Compound ID	Active Site	Jurkat	K562	HT-29	HCT116
**1**	CT-L	54%	63%	62% 90.77 ± 4.76 µM	90% 76.36 ± 3.50 µM
	C-L	19%	28%	76% 79.36 ± 2.38 µM	71% 75.65 ± 2.87 µM
	T-L	16%	NI	NI	NI
**2**	CT-L	72% 73.15 ± 3.18 µM	40%	62% 73.83 ± 1.06 µM	74% 50.92 ± 1.50 µM
	C-L	74% 42.37 ± 2.67µM	63%	69% 62.06 ± 3.90 µM	74% 43.47 ± 2.89 µM
	T-L	56% 93.48 ± 1.16 µM	42%	52% 89.26 ± 1.23 µM	44%
**3**	CT-L	33%	59%	31%	33%
	C-L	25%	39%	19%	NI
	T-L	33%	12%	39%	NI
**4**	CT-L	98% 24.25 ± 5.14 µM	ND	98% 31.30 ± 2.31 µM	98% 35.72 ± 2.60 µM
	C-L	99% 27.05 ± 3.02 µM	ND	82% 35.23 ± 1.56 µM	90% 28.91 ± 1.44 µM
	T-L	NI	ND	NI	NI
**5**	CT-L	64%	ND	81% 84.26 ± 3.78	67% 95.90 ± 12.48 µM
	C-L	36%	ND	29%	28%
	T-L	NI	ND	10%	NI
Bortezomib	CT-L	99% 19.05 ± 1.77 nM	100%	99% 87.36 ± 1.98 nM	99% 80.17 ± 1.78 nM
	C-L	94% 489.4 ± 1.91 nM	97%	99% 640.7 ± 1.82 nM	99% 752.9 ± 1.83 nM
	T-L	41% 3.35 ± 1.90 µM	46%	66% 21.04 ± 1.95 µM	45% 131.35 ± 1.99 µM

**Table 3:** Cell viability assessed through the MTT assay in human cancer cell lines: Jurkat, K562, HT-29, and HCT116. The results are expressed as percentage of cell viability at 100 µM and, when possible, the IC_50_ was determined. Bortezomib was used as positive control. Data presented are mean triplicate value ± SD of at least three independent experiments. Statistical analysis was carried out using one-way ANOVA with Dunnett’s multiple comparisons test. Percentages, SD, and *p* values can be found in Supplementary Material—Tables S8 and S9. ND = Not determined.

	Cell Viability Assays, MTT			
	Cell Line			
Compound ID	Jurkat	K562	HT-29	HCT116
**1**	21% 36.84 ± 5.65 µM	18%	23% 61.71 ± 4.04 µM	24% 65.69 ± 8.16 µM
**2**	77%	60%	65%	89%
**3**	86%	98%	100%	95%
**4**	0% 1.37 ± 0.97 µM	ND	34% 42.96 ± 6.81 µM	0% 1.02 ± 0.55 µM
**5**	0% 43.83 ± 2.27 µM	ND	25% 70.47 ± 1.42 µM	24% 58.41 ± 3.89 µM
Bortezomib	0% 6.48 ± 3.22 nM	0% ND	0% 9.73 ± 2.65 nM	0% 10.70 ± 1.14 nM

## Data Availability

Data are contained within the article and the supplementary materials.

## Supplementary Materials

The following supporting information can be downloaded at: https://www.mdpi.com/article/10.3390/ph16081096/s1, Table S1: Score values (ChemPLP scoring function) obtained in noncovalent docking of known proteasome inhibitors in the three active sites; Table S2: Score values (ChemPLP scoring function, noncovalent docking) of some compounds selected in the SBVS campaign and that were also evaluated in in vitro assays; Table S3: Inhibitory potential of the compounds in isolated 20S proteasomes; Table S4: Inhibitory potential of the compounds in lysates of Jurkat cells; Table S5: Inhibitory potential of the compounds in lysates of K562 cells; Table S6: Inhibitory potential of the compounds in lysates of HT-29 cells; Table S7: Inhibitory potential of the compounds in lysates of HCT-116 cells; Table S8: Percentage of cell viability at 10 µM and 100 µM in Jurkat, HT-29 and HCT-116 cell lines; Table S9: Percentage of cell viability at 10 µM and 100 µM in K562 cell lines; Figure S1: ^1^H NMR (300 MHz, CDCl_3_) of compound **4** (JHG58); Figure S2: HRMS (ESI) of compound **4** (JHG58).
