# Supplementary material for: New Scaffolds of Proteasome Inhibitors: Boosting Anticancer Potential by Exploiting the Synergy of In Silico and In Vitro Methodologies
Source: Pharmaceuticals (Basel). 2023 Aug 2;16(8):1096. doi: 10.3390/ph16081096 (PMC10458307; doi:10.3390/ph16081096)
Supplement: Supplementary file 1 [file pharmaceuticals-16-01096-s001.zip › pharmaceuticals-2521677-supplementary.pdf]

Supplementary Materials

Table S1. Score values (ChemPLP scoring function) obtained in noncovalent docking of known proteasome inhibitors in the three active sites. Besides strong proteasome inhibitors, less active compounds (IC_{50} values for the CT-L activity with a range from 1800 nM to 32400 nM), were also docked and it was possible to verify that those compounds have lower scores than most active compounds.

**Compound ID**	**IC_{50} (nM) – CT-L activity**	**Score values**		
		**CT-L**	**C-L**	**T-L**
Ixazomib	3.4 [35]	81.93	80.10	77.85
Delanzomib	3.8 [44]	83.73	87.30	75.48
TMC-95A	5.4 [45]	81.34	78.62	80.85
Carfilzomib	6 [34]	100.38	95.37	100.32
Bortezomib	7 [34]	73.55	77.85	72.94
Fellutamide B	9.4 [46]	90.77	99.26	94.37
Oprozomib	36 [47]	86.22	87.67	97.13
MG-132	40 [46]	85.34	82.59	85.45
Epoxomicin	47 [48]	80.14	92.28	73.92
Apigenin	1800 [49]	55.12	57.73	52.65
Quercetin	3500 [49]	57.72	59.94	53.87
Kaempferol	10500 [49]	55.81	55.67	51.53
Cyanidin	18400 [50]	51.86	54.44	52.93
Malvidin	32000 [50]	55.05	56.09	54.44
Delphinidin	32400 [50]	49.71	55.65	56.64

Table S2. Score values (ChemPLP scoring function, noncovalent docking) of some compounds selected in the SBVS campaign and that were also evaluated in *in vitro* assays.

**Compound ID**	**Score values**		
	**CT-L**	**C-L**	**T-L**
NSC55467 (compound **1**)	76.87	72.79	70.95
NSC115290 (compound **2**)	79.26	79.10	66.89
NSC728499 (compound **3**)	76.66	79.69	79.13
JHG58 (compound **4**)	69.24	61.47	67.82
Cobicistat (compound **5**)	112.53	106.19	109.13

Table S3. Inhibitory potential of the compounds in isolated 20S proteasomes. The results are expressed as percentage of proteasome inhibition of the CT-L/C-L/T-L activity at 10 µM and 100 µM. Data presented are mean triplicate value ± SD of at least three independent experiments. Statistical analysis was carried out using one-way ANOVA with Dunnett's multiple comparisons test, where ns = p>0.05, *p<0.05, **p<0.01, ***p<0.001, ****p<0.0001 relative to control. ns = not significant.

	**Percentage of proteasome inhibition ± SD – Isolated 20S proteasome**																	
	**CT-L**						**C-L**						**T-L**					
	**10 µM**			**100 µM**			**10 µM**			**100 µM**			**10 µM**			**100 µM**		
**Compound ID**	**%**	**SD**	***p***	**%**	**SD**	***p***	**%**	**SD**	***p***	**%**	**SD**	***p***	**%**	**SD**	***p***	**%**	**SD**	***p***
**1**	0	0	ns	37.72	14.01	****	0	0	ns	54.47	6.73	****	4.43	4.39	ns	51.65	8.35	****
**2**	81.30	4.85	****	98.00	1.21	****	90.77	8.16	****	97.86	0.72	****	0	0	ns	97.00	1.72	****
**3**	8.20	5.53	**	91.40	3.57	****	9.90	3.83	***	88.38	7.02	****	8.80	4.28	****	95.30	3.24	****
**4**	3.30	2.28	ns	69.90	9.61	****	5.80	3.66	ns	77.65	5.17	****	0	0	ns	73.95	6.96	****
**5**	2.85	1.08	ns	35.60	3.95	****	0	0	ns	33.57	4.09	****	5.37	3.81	*	5.35	2.06	ns
Bortezomib	90.95	0.45	****	99.92	0.10	****	33.80	4.25	****	92.80	2.23	****	3.20	2.06	ns	33.58	3.17	****

Table S4. Inhibitory potential of the compounds in lysates of Jurkat cells. The results are expressed as percentage of proteasome inhibition of the CT-L/C-L/T-L activity at 10 μM and 100 μM. Data presented are mean triplicate value ± SD of at least three independent experiments. Statistical analysis was carried out using one-way ANOVA with Dunnett's multiple comparisons test, where ns = p>0.05, **p<0.01, ***p<0.001, ****p<0.0001 relative to control. ns = not significant.

	**Percentage of proteasome inhibition ± SD – Cell lysates (Jurkat)**																	
	**CT-L**						**C-L**						**T-L**					
	**10 μM**			**100 μM**			**10 μM**			**100 μM**			**10 μM**			**100 μM**		
**Compound ID**	**%**	**SD**	***p***	**%**	**SD**	***p***	**%**	**SD**	***p***	**%**	**SD**	***p***	**%**	**SD**	***p***	**%**	**SD**	***p***
**1**	0	0	ns	53.47	2.53	****	2.27	0.94	**	19.43	2.53	****	0	0	ns	16.00	3.87	****
**2**	8.23	1.07	****	72.11	1.84	****	26.87	1.21	****	73.45	2.04	****	7.15	1.92	****	56.17	2.13	****
**3**	0	0	ns	32.80	2.21	****	0	0	ns	25.42	2.50	****	0	0	ns	33.04	2.94	****
**4**	10.49	2.41	****	98.00	0.20	****	8.16	1.90	****	98.94	0.22	****	0	0	ns	0	0	ns
**5**	0	0	ns	63.73	1.42	****	0	0	ns	36.16	1.75	****	0	0	ns	0	0	ns
Bortezomib	99.63	0.55	****	99.30	0.61	****	92.00	0.90	****	94.22	0.99	****	32.04	2.16	****	40.55	2.38	****

Table S5. Inhibitory potential of the compounds in lysates of K562 cells. The results are expressed as percentage of proteasome inhibition of the CT-L/C-L/T-L activity at 10 μM and 100 μM. Data presented are mean triplicate value ± SD of at least three independent experiments. Statistical analysis was carried out using one-way ANOVA with Dunnett's multiple comparisons test, where ns = p>0.05, ****p<0.0001 relative to control. ns = not significant.

	Percentage of proteasome inhibition ± SD – Cell lysates (K562)																	
	CT-L						C-L						T-L					
	10 μM			100 μM			10 μM			100 μM			10 μM			100 μM		
**Compound ID**	**%**	**SD**	***p***	**%**	**SD**	***p***	**%**	**SD**	***p***	**%**	**SD**	***p***	**%**	**SD**	***p***	**%**	**SD**	***p***
**1**	0	0	ns	62.74	2.50	****	0	0	ns	28.18	3.07	****	0	0	ns	4.06	2.03	ns
**2**	13.15	2.95	****	40.15	1.97	****	0	0	ns	63.20	1.87	****	5.12	1.04	****	42.15	1.90	****
**3**	9.34	1.66	****	59.13	1.94	****	14.16	2.10	****	38.81	2.35	****	0	0	ns	12.22	2.11	***
Bortezomib	95.17	1.12	****	99.60	0.61	****	94.99	0.86	****	97.19	0.95	****	36.08	1.76	****	45.45	2.42	****

Table S6. Inhibitory potential of the compounds in lysates of HT-29 cells. The results are expressed as percentage of proteasome inhibition of the CT-L/C-L/T-L activity at 10 μM and 100 μM. Data presented are mean triplicate value ± SD of at least three independent experiments. Statistical analysis was carried out using one-way ANOVA with Dunnett's multiple comparisons test, where ns = p>0.05, *p<0.05, **p<0.01, ****p<0.0001 relative to control. ns = not significant.

	**Percentage of proteasome inhibition ± SD – Cell lysates (HT-29)**																	
	**CT-L**						**C-L**						**T-L**					
	**10 μM**			**100 μM**			**10 μM**			**100 μM**			**10 μM**			**100 μM**		
**Compound ID**	**%**	**SD**	***p***	**%**	**SD**	***p***	**%**	**SD**	***p***	**%**	**SD**	***p***	**%**	**SD**	***p***	**%**	**SD**	***p***
**1**	0	0	ns	62.12	2.93	****	0	0	ns	76.03	2.76	****	0	0	ns	5.14	1.94	*
**2**	2.11	0.94	ns	62.16	1.92	****	6.78	2.32	****	69.25	2.04	****	0	0	ns	52.24	2.10	****
**3**	0	0	ns	31.37	1.90	****	0	0	ns	18.64	2.43	****	0	0	ns	38.92	2.68	****
**4**	10.10	1.95	****	97.93	0.24	****	0	0	ns	82.12	0.93	****	0	0	ns	0	0	ns
**5**	0	0	ns	81.18	1.81	****	0	0	ns	29.29	2.74	****	0	0	ns	10.02	2.30	****
Bortezomib	97.99	0.39	****	99.37	0.40	****	92.93	1.67	****	99.05	0.13	****	50.94	2.55	****	65.97	3.52	****

Table S7. Inhibitory potential of the compounds in lysates of HCT-116 cells. The results are expressed as percentage of proteasome inhibition of the CT-L/C-L/T-L activity at 10 µM and 100 µM. Data presented are mean triplicate value ± SD of at least three independent experiments. Statistical analysis was carried out using one-way ANOVA with Dunnett's multiple comparisons test, where ns = p>0.05, *p<0.05, **p<0.01, ***p<0.001, ****p<0.0001 relative to control. ns = not significant.

	**Percentage of proteasome inhibition ± SD – Cell lysates (HCT-116)**																	
	**CT-L**						**C-L**						**T-L**					
	**10 µM**			**100 µM**			**10 µM**			**100 µM**			**10 µM**			**100 µM**		
**Compound ID**	**%**	**SD**	***p***	**%**	**SD**	***p***	**%**	**SD**	***p***	**%**	**SD**	***p***	**%**	**SD**	***p***	**%**	**SD**	***p***
**1**	0	0	ns	90.18	1.90	****	0	0	ns	71.23	3.10	****	0	0	ns	0	0	ns
**2**	6.93	2.67	***	74.20	3.06	****	11.14	2.95	****	74.08	3.77	****	0	0	ns	44.11	3.10	****
**3**	0	0	ns	33.01	2.88	****	0	0	ns	0	0	ns	0	0	ns	8.10	1.97	****
**4**	22.07	2.25	****	97.99	0.42	****	69.20	2.15	****	90.08	2.73	****	0	0	ns	0	0	ns
**5**	0	0	ns	66.87	2.75	****	0	0	ns	28.15	1.54	****	0	0	ns	0	0	ns
Bortezomib	97.94	0.57	****	99.14	0.15	****	94.21	1.97	****	99.00	0.53	****	26.18	2.84	****	45.05	2.91	****

Table S8. Percentage of cell viability at 10 µM and 100 µM in Jurkat/HT-29/HCT-116 cell line. The results are expressed as percentage of cell viability at 10 µM and 100 µM. Cell viability in control samples was arbitrarily set as 100% and its levels in the tested compounds and/or BTZ-treated samples were plotted as percentage of this value. Data presented are mean triplicate value ± SD of at least three independent experiments. Statistical analysis was carried out using one-way ANOVA with Dunnett's multiple comparisons test, where ns = p>0.05, **p<0.01, ***p<0.001, ****p<0.0001 relative to control. ns = not significant.

	**Percentage of cell viability ± SD**																	
	**Jurkat**						**HT-29**						**HCT-116**					
	**10 µM**			**100 µM**			**10 µM**			**100 µM**			**10 µM**			**100 µM**		
**Compound ID**	**%**	**SD**	***p***	**%**	**SD**	***p***	**%**	**SD**	***p***	**%**	**SD**	***p***	**%**	**SD**	***p***	**%**	**SD**	***p***
**1**	98.66	1.22	ns	21.02	2.45	****	96.67	3.05	ns	23.33	5.50	****	95.52	4.04	ns	24.00	7.73	****
**2**	80.22	3.92	****	77.18	3.60	****	79.89	3.15	****	65.43	3.22	****	97.98	1.03	ns	89.14	3.18	**
**3**	98.10	0.31	ns	85.97	2.20	****	100	0	ns	100	0	ns	100	0	ns	95.25	4.20	ns
**4**	23.29	1.27	****	0	0	****	94.14	3.03	ns	34.27	4.96	****	38.44	4.76	****	0	0	****
**5**	93.33	3.61	ns	0	0	****	98.33	1.53	ns	25.36	5.12	****	98.67	1.16	ns	23.67	6.05	****
Bortezomib	0	0	****	0	0	****	0	0	****	0	0	****	0	0	****	0	0	****

Table S9. Percentage of cell viability at 10 μM and 100 μM in K562 cell line. The results are expressed as percentage of cell viability at 10 μM and 100 μM. Cell viability in control samples was arbitrarily set as 100% and its levels in the tested compounds and/or BTZ-treated samples were plotted as percentage of this value. Data presented are mean triplicate value ± SD of at least three independent experiments. Statistical analysis was carried out using one-way ANOVA with Dunnett's multiple comparisons test, where ns = p>0.05, **p<0.01, ***p<0.001, ****p<0.0001 relative to control. ns = not significant.

	**Percentage of cell viability ± SD – K562**					
	**10 μM**			**100 μM**		
**Compound ID**	**%**	**SD**	***p***	**%**	**SD**	***p***
**1**	93.12	2.15	**	18.23	3.26	****
**2**	86.30	3.61	****	86.52	4.19	***
**3**	100	0	ns	100	0	ns
Bortezomib	0	0	****	0	0	****

Figure S1. ^{1}H NMR (300 MHz, $CDCl_3$) of compound 4 (JHG58).

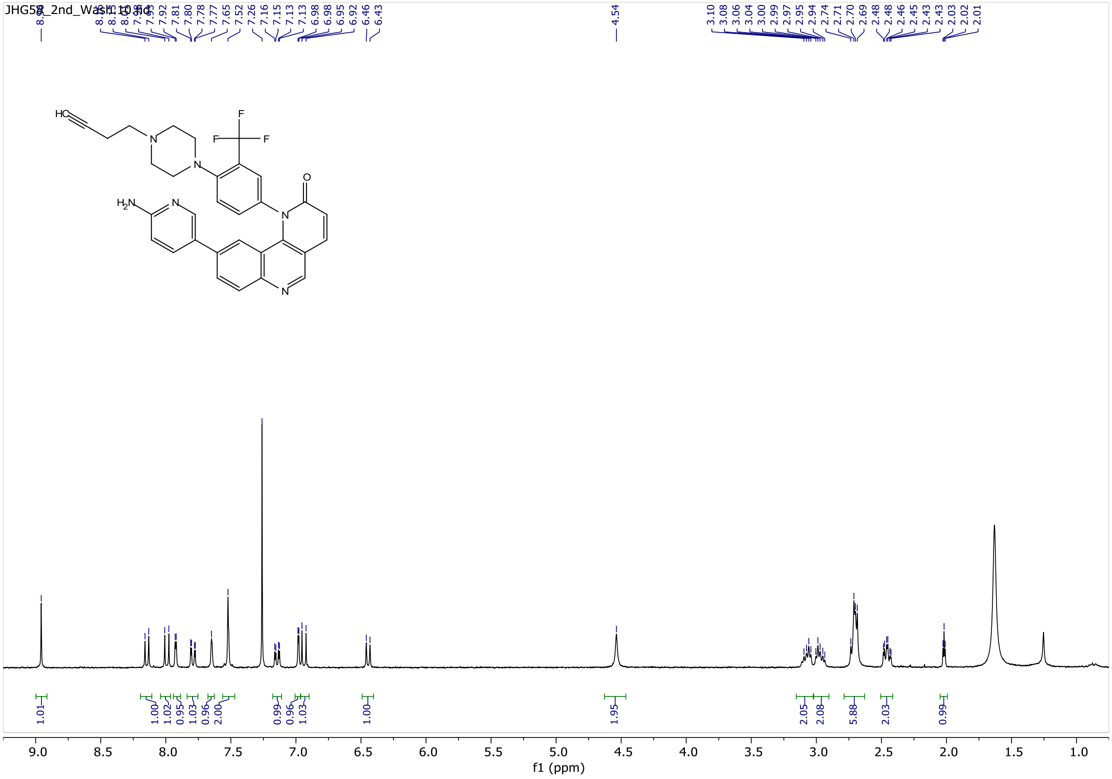

Figure S2. HRMS (ESI) of compound 4 (JHG58).

Sample Name	JHG58	Instrument	impact II	1825265.10036	
Comment					

Acquisition Parameter

Source Type	ESI	Ion Polarity	Positive	Set Nebulizer	2.8 Bar
Focus	Active	Set Capillary	4500 V	Set Dry Heater	200 °C
Scan Begin	100 m/z	Set End Plate Offset	-500 V	Set Dry Gas	8.0 l/min
Scan End	1350 m/z	Set Charging Voltage	2000 V	Set Divert Valve	Waste
		Set Corona	0 nA	Set APCI Heater	0 °C

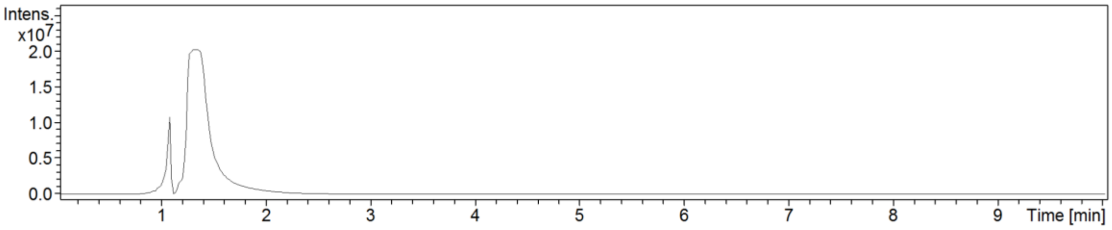

+MS, 1.3min #76

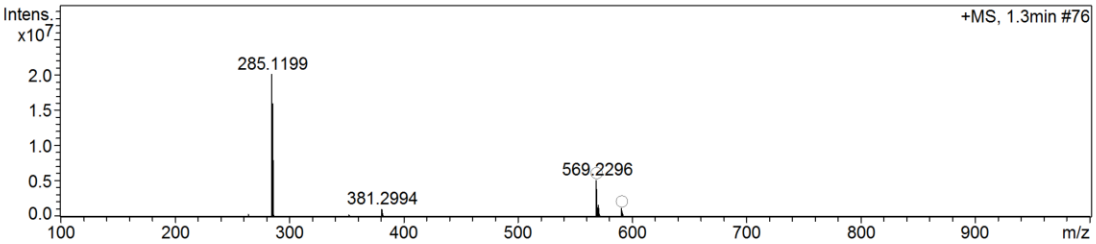

Meas. m/z	#	Ion Formula	m/z	err [ppm]	mSigma	# mSigma	Score	rdb	e⁻ Conf	N-Rule
569.2296	1	C32H28F3N6O	569.2271	-4.3	25.2	1	100.00	21.0	even	ok
591.2109	1	C32H27F3N6NaO	591.2091	-3.0	6.4	1	100.00	21.0	even	ok
